# ZPD Retrieval Performances of the First Operational Ship-Based Network of GNSS Receivers over the North-West Mediterranean Sea

**DOI:** 10.3390/s24103177

**Published:** 2024-05-16

**Authors:** Andrea Antonini, Luca Fibbi, Massimo Viti, Aldo Sonnini, Simone Montagnani, Alberto Ortolani

**Affiliations:** 1 Laboratory of Monitoring and Environmental Modelling for the Sustainable Development (LaMMA), 50019 Sesto Fiorentino, Italy; antonini@lamma.toscana.it (A.A.); viti@lamma.toscana.it (M.V.); montagnani@lamma.toscana.it (S.M.); ortolani@lamma.toscana.it (A.O.); 2 Institute for the Bioeconony (IBE), National Research Council (CNR), 50019 Sesto Fiorentino, Italy; 3 National Institute for Astrophisics, 50125 Florence, Italy; aldo.sonnini@inaf.it

**Keywords:** GNSS meteorology, atmospheric water vapour, ship meteorology, ZPD estimation, ZPD validation, reanalysis weather prediction models

## Abstract

This work presents the design and implementation of an operational infrastructure for the monitoring of atmospheric parameters at sea through GNSS meteorology sensors installed on liners operating in the north-west Mediterranean Sea. A measurement system, capable of operationally and continuously providing the values of surface parameters, is implemented together with software procedures based on a float-PPP approach for estimating zenith path delay (ZPD) values. The values continuously registered over a three year period (2020–2022) from this infrastructure are compared with the data from a numerical meteorological reanalysis model (MERRA-2). The results clearly prove the ability of the system to estimate the ZPD from ship-based GNSS-meteo equipment, with the accuracy evaluated in terms of correlation and root mean square error reaching values between 0.94 and 0.65 and between 18.4 and 42.9 mm, these extreme values being from the best and worst performing installations, respectively. This offers a new perspective on the operational exploitation of GNSS signals over sea areas in climate and operational meteorological applications.

## 1. Introduction

Water vapour plays a major role in atmospheric processes: it is the main agent of the atmospheric greenhouse effect [1], a key factor in the mass and heat exchange between the Earth’s surface and the troposphere, and a crucial term in atmospheric liquid and solid precipitation. Its atmospheric values determine the processes of cloud and precipitation systems formation, constrain evapotranspiration, impact on weather dynamics, and knowledge of the variations in its concentrations and spatial localisation affects the capability of forecasting and nowcasting of severe weather events. Extended measurements of atmospheric water vapour are, thus, essential for climate and meteorology sciences and operational applications [2]. Several methods have been developed for automatic and real-time measurements, ground- or satellite-based, most of which provide detailed information regarding the distribution of moisture, but mainly over land areas. More precisely, satellites can observe homogeneously over land and sea, but passive satellite measurements, in the proper bands for water vapour retrieval, are sensitive to emissions from the upper tropospheric layers [3]. Therefore, there is an observation gap in the lower troposphere, where water vapour normally has the highest concentration. Such a gap can be reduced by ground measurements, but over the sea, measurements are generally very sparse, due to technical limitations and high costs. The knowledge of water vapour content and its space-time dynamics over the sea is very important in territories largely surrounded by the sea, such as the Italian peninsula—the concentration of humidity over the sea is one key driver of intense or extreme precipitation events in coastal and inland areas. This work is directed towards the design, implementation and testing of an operational ship-based infrastructure, as a pilot experiment for the continuous monitoring of atmospheric parameters over the sea.

Atmospheric humidity can be expressed as a quantity integrated along the observing direction and is defined as integrated water vapour (IWV). IWV can be estimated using Global Navigation Satellite System (GNSS) signals. Measurements from GNSS receivers are combined with surface meteorological observations, whose data are processed to obtain the zenith path delay (ZPD), i.e., the delay of satellite navigation signals refracted in the troposphere and projected to the zenith, which mainly depends on atmospheric pressure, temperature and humidity ([4]).

The atmospheric water vapour estimation problem from GNSS measurements can be addressed with different techniques, such as geometric projection of slant delays ([5]), probabilistic inference ([6]), and tomographic approaches [7,8]. A very comprehensive review of GNSS meteorology theory and methods is provided in [9], where several related applications are described.

Through extensive investigation, it has been found that the precision of ZPD estimations using GNSS equipments is strongly dependent on the possibility of modelling certain ancillary information, such as the precise satellite ephemeris, clock parameter estimations [10], and ocean tide loading [11]. ZPD values are systematically provided by the International GNSS Service (IGS) center for reference stations around the world, together with other products needed for their computation, such as precise combined final orbits and clocks of the different constellations [12]. Such ancillary features are mandatory to process GNSS data using the most recently introduced processing technique called precise point positioning (PPP) based on a single receiver and the observations of as many constellations as possible (Multi-GNSS) to increase the precision of the estimates of the various parameters [13,14]. An exhaustive introduction to PPP is provided in [15,16], with a detailed mathematical formulation of the GNSS data processing, including the critical issue of ambiguity resolution of the signal phase observations. A very interesting comparison between different GNSS PPP processing software packages and related positioning accuracy is provided in [17], where a series of open-source software and online services are analysed with an extended dataset of 45 IGS stations, showing the capability of most of the PPP procedures to provide millimeter level accuracy in position determination. The positioning accuracy of the PPP approach is, however, strongly related to the accuracy of other parameters, including ZPD.

The main goal of the present work is to set up an operational network of GNSS-based water vapour measurements from moving ships, over the north-west Mediterranean, an area of storm generation, to use in nowcasting and forecasting activities (through data assimilation procedures). The widespread observation system consists of Multi-GNSS receivers and weather stations installed on eight liners operating in the Mediterranean. For data processing, we adopted the PPP approach, which combines high accuracy with the possibility of separately processing data for each receiver. One of the main problems in the PPP approach is the time needed for the algorithm to converge in the ambiguity resolution, especially for float ambiguity choice. This results in a convergence time of approximately 15–30 min, from the first processed observation epoch, in the absence of external constraints for the ionospheric and tropospheric path delays [18]. This aspect is particularly relevant in challenging applications where moving systems, like those on board ships, can cause further increase in the convergence time and further fluctuations of the observations. Pioneering tests on GPS meteorology measurements from ships were undertaken in [19,20], while some experiments over the Mediterranean area are described in [21]. In a research project conducted in the Baltic sea, the use of multi-constellations as possible means of improving measurement precision was examined [22]. A later experiment on the use of GPS receivers on board a ship for atmospheric IWV estimations was conducted during a two-month cruise in the equatorial Indian Ocean ([23]). The behaviour of hardware and software equipment was also evaluated during an Indian Ocean dipole event. As expected, errors from ship-based retrievals were significantly higher than those from fixed stations due to the unknown of the receiver position at any epoch. The results of the validation analysis, using ancillary data as reference and also including radiosonde launches, are given in [24]; the values of GPS-based IWV obtained were in agreement with the reference ones (rms errors less than 2.5 mm). Collection of ship-based GNSS meteorology observations was also recently conducted over the Atlantic Ocean, in the Barbados region, and in the Indian and Austral Oceans, as described in [25] and [26], respectively, highlighting the importance of this kind of measurement for climatological and meteorological applications. Finally, in [27], a very large dataset of shipborne GNSS-based ZPD measurements collected during six voyages all around the globe was discussed and validated. However, the test periods of the experiments were limited to a few weeks or a few months, so the potential application of the results obtained is limited with regard to longer time periods in different seasons and different types of meteorological situations.

This paper provides for the first time a very large dataset (over more than three years) of automatic, continuous measurements, along different routes (so at different points) in the Mediterranean Sea, at the same time and in different weather conditions, spanning the seasons. The design and implementation of the infrastructure, including the hardware and software specific solutions, are described from both a technical and a scientific point of view in Section 2. A validation analysis, using as reference the ZPD values provided by a numerical weather reanalysis model, was conducted for the full 3-year dataset period. This approach partly recalls other previously developed works, such as [28], that involve comparison between ZPD computed with data from a set of globally distributed ground GNSS stations and with numerical weather prediction reanalysis, respectively. The important original contribution of the present work is that the proposed system is operational and was for the first time validated at sea. Section 3 presents the results of the measurements and a comparison with a long series of model reanalysis reference data, evaluating the error parameters of the ZPD estimation. Section 4 provides an analysis of the results with detailed discussion of specific behaviours observed in the different stations. Finally, in Section 5, comments on the study and the conclusions are provided.

## 2. Materials and Methods

The infrastructure described in this work consists of a number of GNSS meteorology stations, most of them (eight systems) installed on board ferries operating in the north-west Mediterranean Sea (mainly in the Ligurian and Tyrrhenian Seas), and two installed on fixed inland points, namely, in the towns of Grosseto and Sesto Fiorentino (Florence) in Tuscany (Italy) [29] (see Table 1 for a complete list). Measurements were gathered from different (and mostly non-fixed) points, but were sent to a unique system node to be jointly processed to produce the ZPD products. In this section, the system architecture is described, detailing the implemented hardware and software solutions.

### 2.1. Ship-Based GNSS Meteorology System Architecture

The system on board each ship was equipped with a commercial geodetic station for GNSS signal detection and with a weather station. A 3D choke ring GNSS antenna able to significantly mitigate signal multipath effects was connected with a scientific receiver with a capacity of more than 500 receiving channels. The antennas were installed in a high ship position in order to be as free as possible from obstacles along the signal paths, making artificial signal reflections negligible for non-horizontal (or non-sub-horizontal) paths. The receivers were installed in environments protected from atmospheric agents. These GNSS stations were able to simultaneously detect signals from all global satellites constellations (i.e., Galileo, GPS, GLONASS, BeiDou).

**Figure 1:**
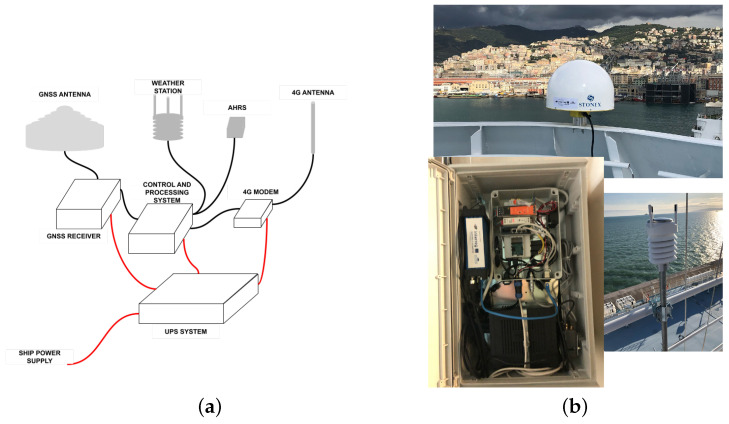
GNSS meteorology equipment onboard ships. (**a**) System architecture, (**b**) Photos of GNSS antanna (**top**), GNSS receiver and data collector (**bottom left**), weather station (**bottom right**).

Surface meteorological observations were collected using an automatic system comprising a weather station and an attitude heading reference system (AHRS). The latter used an inertial measurement unit (IMU) consisting of micro-electro-mechanical system (MEMS) inertial sensors to measure the angular rate, the acceleration, and Earth’s magnetic field to derive an estimate of the ship’s attitude. The heading and speed of the ship were then used at any time to transform relative (to the ship) wind vectors to absolute wind vectors, i.e., relative to the Earth’s reference system. The set of atmospheric measurements was completed by collection of surface pressure, temperature, humidity and precipitation rate (both rain and hail) data. The processing and control system acquired all the measurements collected from the GNSS receiver and the weather station, undertook some preliminary processing and sent the data to the ground center through a mobile communication 4G system, consisting of a router and an external omnidirectional antenna. Both weather data (in a simple text format) and GNSS receiver data (in standard Receiver INdependent EXchange: RINEX format) were aggregated hourly and transmitted in real-time, or quasi real-time, according to the spatial coverage of the 4G cellular network connectivity. A schematic diagram of the system architecture is shown in Figure 1.

### 2.2. Processing Software

#### 2.2.1. Onboard Pre-Processing

The control and processing system was managed by a mini-PC interfaced with the other equipment in different ways. Two USB/RS485 converters were connected with the weather station and the AHRS, respectively. An Ethernet port was used to connect the GNSS receiver to obtain the data and to a 4G router for Internet connectivity.

The software for the onboard instruments and data management was a dedicated C++ code designed using a multi-task and multi-thread approach. The main process of this software managed the following independent threads:

- GNSS time and position data acquisition;
- Weather station data acquisition;
- AHRS data ingestion;
- Data publication (on a local HTML server) and storage.

A child process was run for retrieving the AHRS data. The GNSS receiver automatically stored internally the data received by different satellite constellations, and created aggregated RINEX files. The aggregation period could be selected; for reasons of synchronisation with other cascade processing (e.g., the assimilation into numerical meteorological models), we opted for a one-hour period. Finally, a third synchronised process sent the RINEX and surface weather data by FTP to the central ground server for successive processing.

#### 2.2.2. ZPD Retrieval

Among the available software packages to support the processing of RINEX data, we opted for MG-APP (Multi GNSS Automatic Precise Positioning) [30], that was found to be accurate, reliable and efficient. MG-APP is an open source software developed to process multi-constellation GNSS data from GPS, GLONASS, BeiDou and Galileo platforms, both in static and in kinematic mode. It provides different data-smoothing solutions, mainly based on a Kalman filter and a square root information filter (SRIF). Moreover, the back smooth filtering approach allows to apply the filter in both forward and backward modes through the observation epochs, limiting the effects of the convergence delay to reach a stable ambiguity solution, typical of float-PPP approaches. The processing flow also contains a check of the control values that are labelled and corrected or eventually rejected if outside a range of admitted values. The analyses in this work were performed using version 1.0 of the MG-APP software, freely available at [31].

The processing scheme used was the float-PPP (precise point positioning), separately applied to each receiver, where “float” means that the ambiguity solution is not constrained to an integer value, to account for other error sources. This approach allows to achieve position accuracy at the centimeter level for static equipment or at the decimeter level for “slow-moving” equipment, such as on board a commercial ship. The possibility to access and modify the software code allowed making some essential changes for operational and optimised use in the ZPD estimation. The main upgradings that we performed on the MG-APP are listed below.

- We introduced a command line interface for automatic execution inside of the script suitable for near-real-time processing, while the original software was designed for interactive use only trough a GUI (graphic user interface).
- We included the capability to process different types of input files, namely observation and navigation RINEX (obs, nav), orbits, clocks and Earth rotation corrections (sp3, clk, erp), antenna calibration (ant), ocean tidal loading (blq, otl), and tropospheric gridded corrections (grd), regardless of their name or extension.
- The possibility of processing data coming also from the QZSS (Quasi-Zenith Satellite System) constellation.
- We added the capability to perform ocean tide calculation using gridded input data. The software originally used only site-specific input data for the ocean tide model, while the modification allowed the interpolation of data contained in a grid file. The ocean tide gridded model used was the FES2004 [32]. Both the ocean tide grid model and the interpolation procedure were implemented as in the GAMIT-GLOBK 10.71 software [33]. In this way, the ocean tide model can also be applied to moving systems, such as ships in our case.
- The original version of the MG-APP software performed a time interpolation of the satellite clocks corrections to match all the observation epochs. Unfortunately, such interpolation introduces an error that should be computed and corrected, for example, through a stochastic model of satellite clock interpolation errors, as proposed in [34]. In our implementation, satellite clock signals were not interpolated. Instead, the processing involved only the epochs for which satellite clocks corrections were available. This brought benefits especially in the near-real time processing, where the ZPD computation was available every 15 and 5 min using ultra-rapid and rapid orbits, respectively. It was found that considering observation inputs only when the satellite clocks were available led to significant improvement in the final results, even if less output data were produced. On the other hand, when using the final ephemeris available after a few days (from 12 to 19), the frequency of satellite clock correction was 30 s, so very close to the frequency of observations. The use or not of interpolation on the clock correction was consequently not relevant.

Many processing parameters were left at their default values [30]. The cut-off of the satellite observation angle was 10 degrees to achieve higher mitigation of reflections and interference, according to [35], which was valid for processing in the absence of weighting functions of satellite elevation. The ZPD values used in this work were obtained using the final orbits produced by CODE (Center for Orbit Determination in Europe) and downloaded from [36]. Specifically, the multi-GNSS orbit and clock solution (COM) were used, produced by the CODE analysis center for the IGS within the framework of the Multi-GNSS Experiment (MGEX). The selected products contained the position and clock of the GPS, GLONAS, Galileo, QZSS and BeiDou satellites at a resolution of 300 s for the orbits and 30 s for the clock [37]. The implemented processing system on a daily basis produced the ZPD at a 30 s time interval (the same as clock solutions) for each GNSS station whether fixed or moving (as listed in Table 1). The Saastamoinen model [38] was used in combination with the mapping function VMF1 (Vienna Mapping Function 1) [39] for the first guess estimation of the tropospheric hydrostatic delay. The SRIF recursive filter was adopted for random walk estimation of the zenith wet delay (ZWD): the first guess associated variance was 10 m, and the subsequent time variance was about 2 mm h^−1/2^. The surface air temperature and pressure needed by the Saastamoinen model were given by the meteorological stations installed close to the GNSS receivers in each ship-based or fixed installation. When occasionally missing, such information was derived from GPT 3.5 gridded data (Global Model of Pressure and Temperature) [40]. All ancillary data were automatically downloaded during the processing.

### 2.3. Validation with NWP Data Reanalysis

First, we have to stress that no reference-independent ZPD measurements exist that provide data at the same time and location of our moving instruments. To overcome this problem, the vertical profile extracted from the weather models can be used. A dataset was selected from the Modern-Era Retrospective Analysis for Research and Applications, Version 2 (MERRA-2) [41], as these weather model reanalyses represent one of the most complete and reliable reconstructions of the atmospheric state in any place and at any past time in the reanalyses domain.

#### 2.3.1. Merra-2 Reanalysis Data

MERRA-2 is a long-term reanalysis dataset implemented by the Global Modeling and Assimilation Office (GMAO) of NASA that provides data beginning in 1980. It is an evolution of the previous MERRA dataset that includes an assimilation module for satellite hyperspectral, microwave and GNSS radio occultation observations in addition to ground weather stations, radar and balloon data, already assimilated by the previous version. The Earth atmospheric domain is organised in a regular lat–lon horizontal grid with 576 points in the longitudinal direction and 361 points in the latitudinal one, corresponding to a resolution of 0.625° × 0.5°. Quantities are provided in the vertical direction on either the native vertical grid (at 72 model layers or the 73 edges), or interpolated to 42 reference pressure levels. For this work, we selected the latter option, using the product named M2I6NPANA, as downloaded from [42], consisting of 6-hourly instantaneous 2D fields of sea level pressure (SLP) and surface pressure (SP), 3D meteorological fields, geopotential height (HGT), ozone mixing ratio (OMR), specific humidity (QV), air temperature (T), eastward wind component (U), and northward wind component (V) at 42 pressure levels (P) from 1000 hPa to 0.1 hPa. As extensively demonstrated in the scientific literature, ZPD is directly provided by the refractivity integrated along the vertical direction [43,44]. Therefore, for our purposes, i.e., to derive reference ZPD values from MERRA-2 data, the relevant data were SLP, SP, P, HGT, QV and T, at all the available vertical levels.

#### 2.3.2. Comparison Methods

On a daily basis, the MG-APP software processes GNSS observations collected every second, while the output ZPD values have a time frequency of 30 s. Such ZPD values are further filtered to eliminate outliers. ZPD physically plausible values are between 1800 and 2800 mm [45,46], so this range was the one adopted in the filtering phase.

The values of ZPD were obtained from the MERRA-2 data by discrete piecewise linear integration (i.e., sum) of the refractivity, for any selected pixel, along the vertical profile given by the 42 pressure levels, as described in [6]. Note that since MERRA-2 data do not contain the surface values of T, QV and HGT, these were estimated through a linear extrapolation or interpolation, when feasible, of the values at the closest vertical levels.

**Figure 2:**
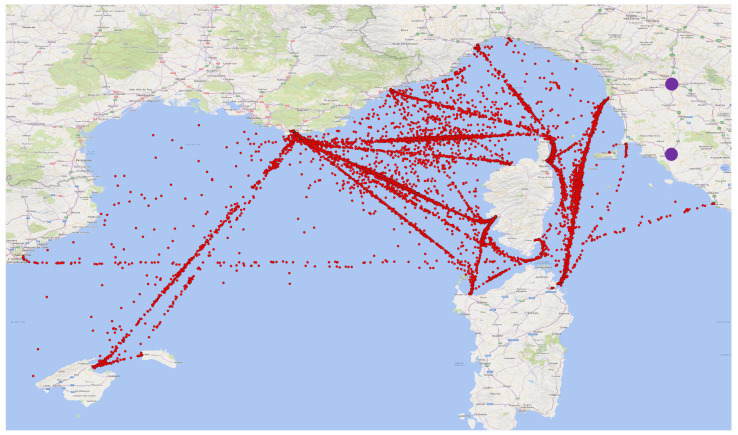
Points (red) available for the comparison between GNSS-based and MERRA-2-based ZPD over the period 2020–2022. The two purple circles show the positions of the fixed stations.

The comparison between the observations and model-derived data started from the spatial and temporal matching. The model pixels were selected according to the ship position at any observation time. As the MERRA-2 data are provided at synoptic time, only a very limited number of perfect pairs matching is possible, each six hours. So the MG-APP ZPD data were further processed to extract the average over a one-hour window centred on the synoptic time (i.e., 30 min before and 30 min after). The resulting points available for the comparison are visible on Figure 2, where the main ship routes clearly emerge as the most densely pointed areas. However, there are also a high number of points outside of these main routes, which show how the measurement dataset from a ship is much wider regarding the spatial coverage.

## 3. Results

ZPD values from ship, measured by means of the MG-APP software applied to the RINEX from the onboard equipment, were compared with the ones derived from MERRA-2 vertical profiles over the ship positions. The main results of this comparison are shown in this section, for the whole three year period 2020–2022.

### Description of ZPD Values

**Figure 3:**
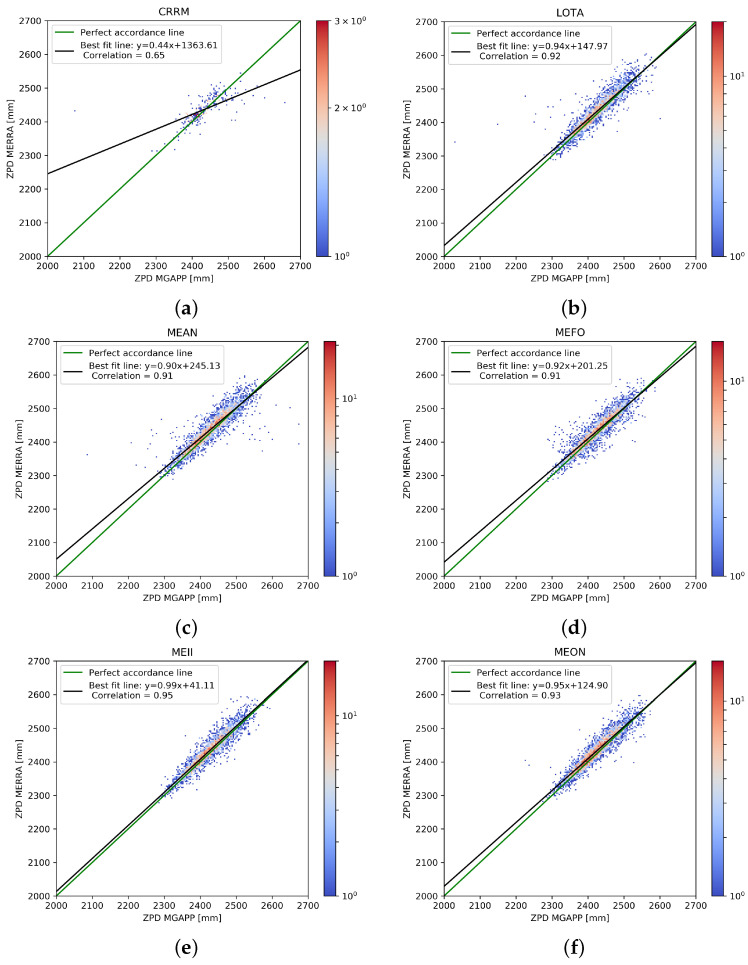

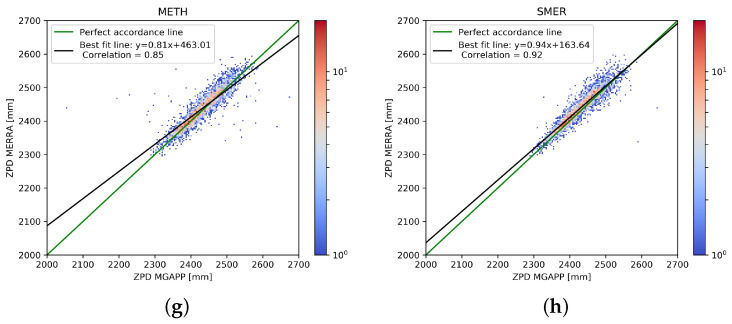
Density scatter plots of GNSS-ZPD (using MG-APP software) vs. ZPD retrieved from MERRA-2 reanalysis data. Only data from ship stations are included in this figure. All available data in the period 1 January 2020 and 31 December 2022 are considered.

**Figure 4:**
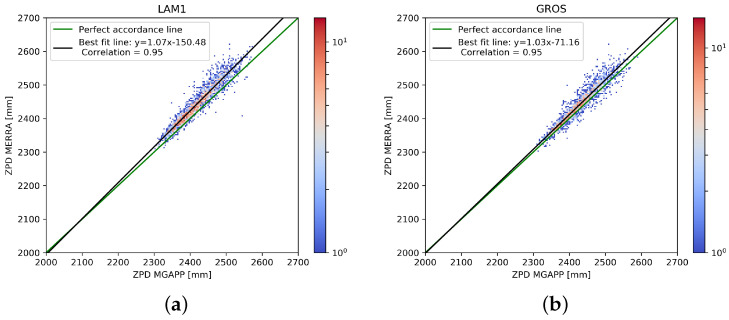
Density scatter plots for GNSS-ZPD (using MG-APP software) vs. ZPD retrieved from MERRA-2 reanalysis data for the two fixed stations. All available data in the period 1 January 2021 and 31 December 2022 are considered (i.e., one year less than for the ship data of Figure 3).

In Figure 3 and Figure 4, the density scatter plots provide an overview of the performance of the ZPD estimation. For all considered stations, a strong linear relationship between the estimated ZPD values and corresponding MERRA-2 ones was clearly found. Moreover, the areas of higher density were very near to the perfect accordance line. The dispersion varied sometimes significantly from ship to ship; this can be reasonably ascribed to a restricted number of causes, such as the adopted installation solutions, interference with other onboard equipment, and ship movements. The number of persisting outliers was also rather high for a few stations (e.g., CRRM, METH, MEAN), where the best-fit line, i.e., the straight line fitted to the data through the least square regression approach, deviated from the perfect accordance line.

As expected, the higher was the number of outliers and their scatter, the wider was the deviation of the best-fit line from the perfect one. As also expected, the dispersion was strongly reduced for fixed stations, reported as reference (Figure 4). Note that data for the fixed stations are available for a shorter period (years 2021 and 2022) than for the ships.

**Figure 5:**
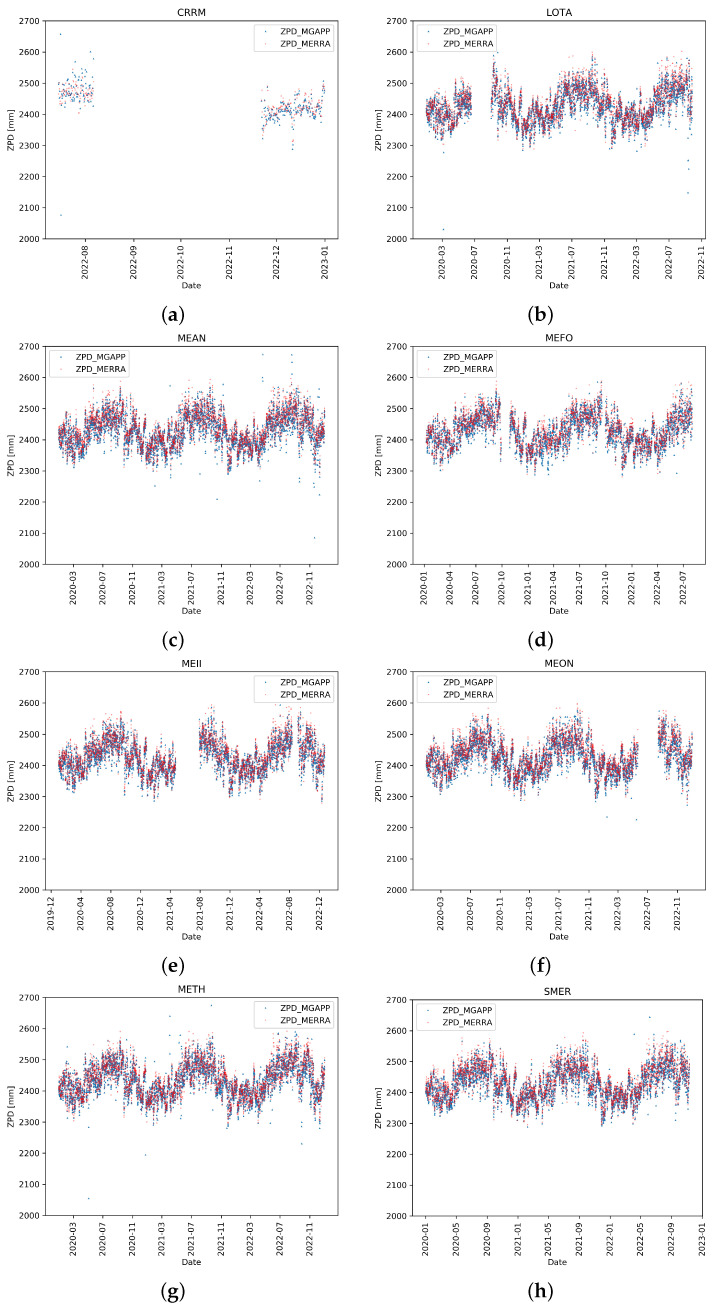
GNSS-ZPD (using MG-APP software). Only ship stations are considered in this figure. All available data in the period 1 January 2020 and 31 December 2022 are considered.

**Figure 6:**
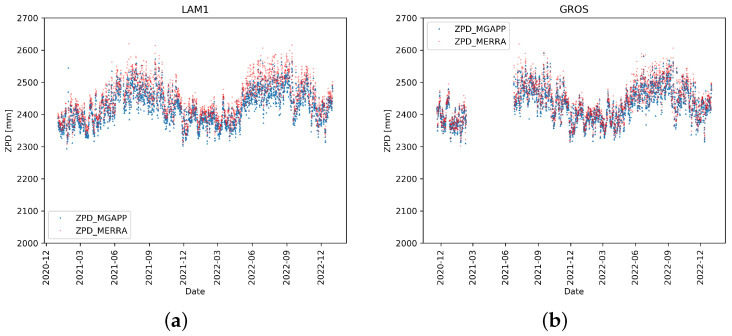
GNSS-ZPD (using MG-APP software) for the two fixed stations. All available data in the period 1 January 2020 and 31 December 2022 are considered.

Figure 5 and Figure 6 allow a further understanding of the behaviour of the different systems. For example, in the case of CRRM, there are two distinct periods with different performances. In the first data sequence, during July 2022, the ZPD was estimated very often to be quite different from the MERRA-2 reference values. This was probably due to an installation issue; in fact, during the receiver stop period (corresponding to the no data in the plot), some interventions because of malfunctioning were carried out, including a new set up of the system, that solved the problem, as is clearly visible by the behaviour for the following period.

With regard to the data for the other ships, the following considerations are relevant. These graphs show ZPD values at only four time points of each day (00, 06, 12, and 18 UTC), due to the already mentioned limited occurrence of MERRA-2 data, so a more accurate reconstruction of the time patterns of the ZPD cannot be provided. However, intra- and inter-daily fluctuations are evident, that overlapped with longer, seasonal, periodic trends.

The seasonal pattern of ZPD is in good accordance for all ship measurements, showing maxima during the summer season and minima during the winter one, reflecting a combination of both atmospheric total pressure trends and water vapour concentration variations. The physical origin of this behaviour is known and is primarily due to the trend of the zenith wet delay (ZWD), as highlighted in several works, such as [47], showing the climatology of integrated water vapour in the European regions, or as in [48], investigating the characterisation of seasonal and diurnal cycles of ZPD at the global level.

We can note that in this context the outliers are quite evident, as almost all of them are outside the main “stripe” of values (i.e., from the variance of the seasonal climatology), and, thus, could be easily identified for removal using additional filters with relatively simple rules.

A quantitative evaluation of the ZPD estimation can be obtained through direct comparison with MERRA-2 values, using as the score parameters, the (Pearson) correlation, the mean square error (MSE), the root mean square error (RMSE) and the mean bias error (MBE). These parameters were computed separately for each station and are shown in Table 2, together with the number of points available for their calculation (during the full three year period) and with the equation of the best-fit line.

As expected, the errors from the ship-based receivers were greater than those from the fixed stations, which also showed a higher correlation value. In fact, the motion of the station on the sea decreased the estimation performances, resulting in higher oscillations of the ZPD values, but only slightly.

As is evident even just from the scatter plots, for all the stations, the correlation value was rather high, always higher than 0.85, with the exception of the CRRM station, which, as we have shown, had some known problems in the first period of operation. Most stations showed RMSE and MSE values that were rather reduced. Only the CRRM and METH ship stations had higher errors due to the higher number of outliers, but within the acceptance window of our general filtering process (i.e., non-upgraded with seasonal constrains). What happened for the fixed station LAM1 was different, as the correlation was very high, 0.95, equal to the correlation that occurred in the other fixed station (GROS), but the values of RMSE and MSE were close to the METH station. Moreover, in Figure 4 and Figure 6, the values of ZPD computed from the GNSS stations (in particular LAM1) were slightly underestimated (i.e., biased) compared to the reference values. This was also confirmed by the high absolute value of MBE, the largest of all. An important bias was also recorded in the second fixed station, GROS.

## 4. Discussion

The beginning of this work dates back to 2010, when a small number of initial experiments testing the feasibility of GPS meteorology applications from ships was available (e.g., [20,24]). We approached the concept of measuring water vapour over marine areas using GNSS signals from ship-based receivers as the main content of a successful FP7 proposal named COSMEMOS [49]. There was an inherent contradiction in seeking to use a mobile station to retrieve atmospheric parameters that are essentially retrieved from the errors in the positioning produced by the atmosphere, measured from receiver stations of well-known positions (i.e., geographic coordinates, including height), such as for fixed stations, installed on stable grounds. Thanks to the simultaneous availability of GNSS measurements from many constellations, in addition to the upgraded accuracy of present systems, we can say that, nowadays, GNSS meteorology instruments can be used operationally on board ships to accurately measure ZPD in whatever conditions, as testified by the results of three years of operational data acquisition, as we have shown.

As explained, measurements were compared with independent data that we considered as reference, i.e., ZPD values derived from the atmospheric parameters provided by the MERRA-2 reanalysis products. It is known that reanalysis data are very accurate as they are reconstructed ex post assimilating as much information as possible from different observations, from in situ, airborne and satellite platforms. Therefore, we can be confident in the reliability of the reference data, while keeping in mind that they provide data on grid points that refer to an area (or better to a flattened volume) and not to a dimensionless point (we note that a MERRA-2 pixel has a horizontal dimension greater than 65 × 50 km^2^).

The results of the comparison highlight a very strict agreement between the quantities, with skill scores comparable with the two other fixed GNSS stations. Moreover the quantitative assessment of the ZPD estimation showed a difference of about 15–30 mm that is consistent with the differences reported in [21,27]. A relevant part of the remaining difference was still due to the presence of outliers that could be further removed considering seasonal acceptance ranges instead of a constant one. The present results and the identified room for improvement enable us to say that mobile equipment provides performances so close to fixed ones that their variability with respect to the MERRA-2 derived values is intrinsic to the differences in the data we are comparing, that we stress again reflect in situ high sampling rate measurements vs. gridded model data. In other words we infer that such differences are in large part real (and correct), coming from the intrinsic (somehow artificial) smoothness of the model parameters, mainly because of their limited space and time effective resolution, and from some errors in the model values and their synchronisation with the real atmospheric state and its dynamics, that even the reanalysis process can never fully cancel.

Therefore, the system showed high precision, accompanied by robustness and reliability, demonstrated during the long period of operation that was analysed. In addition, the use of these systems at sea for several years, subject to critical atmospheric agents, was a real stress-test that we can say was fully passed, as over the time, the performances of the systems were absolutely stable.

As demonstrated, the stations installed on board ships showed relevant fluctuations in the ZPD values and also a greater occurrence of outliers, though particularly on some ships (CRRM, METH, MEAN). The opposite occurred for fixed stations where the dispersion of values was very limited, with very high correlations around 0.95. On the other hand, the MBE was greater, showing a systematic small underestimation error. The cause of the higher MBE values on the fixed station can be identified by considering that the ZPD values are strongly dependent on the atmospheric pressure value at the station level. In the model, the pixels over the sea, where the surface height was constant, pressure values were quite homogeneous, all over the area inside the pixel, and were, therefore, directly comparable with a single point. The same does not hold for the ground pixels, where the presence of non-negligible orographic effects occurs within the pixel, influencing the average surface pressure value, which, as a consequence, can not be representative of a single point. Therefore, it can be hypothesised that this bias is mainly attributable to the average elevation of the terrain in the MERRA-2 ground pixels. This effect was particularly accentuated in the LAM1 station located in Sesto Fiorentino near Florence, at a height of a few tens of meters but on the slopes of a mountain with an over 900 m high summit in the central Italian Apennines chain.

## 5. Conclusions

This work describes the implementation and operation of an infrastructure of GNSS meteorology sensors, consisting of multi-constellation receivers and weather stations, installed on board a series of ferries operating in the north-west Mediterranean Sea (mainly in the Ligurian and Tyrrhenian Seas). The observations were processed by the MG-APP software system that was used in the best possible conditions regarding knowledge of the satellite orbits, i.e., using the final ephemeris, which are the most precise, but available with a delay of about two weeks. This was to allow the assessment of the complete potential of the system by considering the best ZPD estimates usable for scientific (e.g., climatological) studies, but not reachable at the moment in real-time application. This means that for real-time applications, the resulting considerations should be adapted to real operating conditions, i.e., considering the real-time available orbit and clocks and updating the software configuration, as suggested in some reports (e.g., [35]). However, apart from the use of the best ephemeris values, the remainder were as available as in an operational context.

A comparison of the measurements with a reliable global reanalysis model, namely the MERRA-2, supported the use of this infrastructure, as the values were in very significant agreement with the reference values, and very similar to those obtained for the two fixed stations that were included in the analysis. This infrastructure is a promising tool for monitoring water vapour patterns, from climatological trends to fast dynamics in meteorological events, using measurements over the sea that are normally very sparse. This network can, in fact, using a high sampling rate, record the trend in the ZPD, a parameter strongly dependent on the vapour content in the atmosphere, which is the most abundant and variable greenhouse gas and a critical climatic feedback variable. At the same time, it is also a crucial input in numerical weather models because it is the main player in the mass–energy exchange between the Earth’s surface and the troposphere, including precipitation phenomena and their extreme manifestations. The performances of such a measurement system (integrated water vapour between 2.3 kg m^−2^ and 4.6 kg m^−2^) are also compatible with the requirements of [50] both for nowcasting, numerical meteorological prediction and climatological applications.

One of the main limitations of the proposed system is the convergence time for achieving reliable ZPD values. In some receivers (such as METH), we experienced some outliers and anomalous oscillations in the very initial period of processing. This was probably due to the time needed for convergence to ambiguity resolution. Future work should investigate the concatenation of successive days of processing. This should reduce, by a smoothing approach, the occasionally anomalous oscillations in ZPD values due to the transition from one daily processing step to the next.

There is also the possibility that these issues will be reduced by technological innovation and improvement in the performance of processing software, as well as by the growing number of satellites simultaneously tracked by each receiver.

We are planning further analyses to compare these and new data from the network with other reanalysis products and with radiosounding data from ships. At the same time, we will perform some tests to understand if the outlier occurrence in some equipment is due to the solutions adopted for the installation of the apparatus, very often resulting from ship-specific constraints, for example, due to the proximity to other operating instruments that can cause interactions, such as the presence of reflective surfaces near the antenna.

## Figures and Tables

**Table 1:** List of stations.

Station Name	Type of Station	Name of Ship/Site	Lat; Long; Height
MEAN	SHIP	Mega Andrea	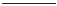
LOTA	SHIP	Pascal Lota	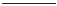
SMER	SHIP	Mega Smeralda	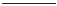
MEFO	SHIP	Mega Express Four	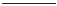
METH	SHIP	Mega Express Three	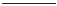
MEII	SHIP	Mega Express Two	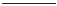
MEON	SHIP	Mega Express	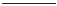
CRRM	SHIP	Cruise Roma	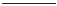
GROS	GROUND	Grosseto	42.760; 11.115; 31 m
LAM1	GROUND	Sesto Fiorentino	43.819; 11.202; 59 m

**Table 2:** Estimation scores. Fixed stations (the two last ones) are labelled in italic.

Station Name	Number of Available Points	Correlation	RMSE [mm]	MBE [mm]	Best Fit Line y = ax + b
MEAN	4376	0.91	24.15	−8.8	y = 0.90 x + 245.13
LOTA	3676	0.91	22.7	−7.9	y = 0.94 x + 147.97
SMER	4175	0.92	22.5	−9.0	y = 0.93 x + 163.63
MEFO	3572	0.91	22.6	−7.5	y = 0.92 x + 201.25
METH	4371	0.85	30.1	−5.8	y = 0.81 x + 463.01
MEII	3857	0.94	18.4	−7.3	y = 0.98 x + 41.1
MEON	4033	0.93	20.3	−8.4	y = 0.95 x + 124.89
CRRM	252	0.65	42.9	−1.1	y = 0.44 x + 1363.61
*GROS*	2549	0.95	21.2	−12.6	y = 1.03 x − 71.16
*LAM1*	2888	0.95	30.4	−24.5	y = 1.07 x − 150.48

## Data Availability

Data are contained within the article.
